# GPHB5 Is a Biomarker in Women With Metabolic Syndrome: Results From Cross-Sectional and Intervention Studies

**DOI:** 10.3389/fendo.2022.893142

**Published:** 2022-06-09

**Authors:** Ting Xiang, Siliang Zhang, Qinge Li, Ling Li, Hua Liu, Chen Chen, Gangyi Yang, Mengliu Yang

**Affiliations:** ^1^ Department of Endocrinology, the Second Affiliated Hospital, Chongqing Medical University, Chongqing, China; ^2^ The Key Laboratory of Laboratory Medical Diagnostics in the Ministry of Education and Department of Clinical Biochemistry, College of Laboratory Medicine, Chongqing Medical University, Chongqing, China; ^3^ Department of Pediatrics, University of Mississippi Medical Center, Jackson, MS, United States; ^4^ Endocrinology, School of Biomedical Science (SBMS), Faculty of Medicine, University of Queensland, Brisbane, QLD, Australia

**Keywords:** GPHB5, bioinformatics, metabolic syndrome, cross-sectional study, intervention study

## Abstract

**Background:**

Animal studies have found that GPHB5 has a similar effect on system metabolism as TSH. However, the relationship between GPHB5 and metabolic diseases remains unknown. This study investigates the relationship between GPHB5 and MetS in young women.

**Methods:**

Bioinformatics analysis was undertaken to explore the relationship between GPHB5 and metabolic-related genes and signaling pathways. EHC and OGTT were performed on all individuals. Lipid-infusion, physical activity, and cold-exposure tests were performed on healthy individuals. Serum GPHB5 concentrations were measured by an ELISA kit.

**Results:**

PPI network showed that 11 genes interacted with GPHB5, in which POMC and KISS1R were involved in glucose and lipid metabolism. GO analysis showed 56 pathways for BP and 16 pathways for MF, in which OPRM1 and MCR families were related to energy metabolism. KEGG analysis found that GPHB5 is associated with lipolysis and neuroactive ligand-receptor interaction pathways. The levels of circulating GPHB5 were significantly increased, while serum adiponectin levels were lower in MetS women compared with healthy women. Obese/overweight individuals had lower adiponectin levels and higher GPHB5 levels. Circulating GPHB5 levels were positively correlated with BMI, WHR, blood pressure, FBG, 2 h-BG, HbA1c, FIns, 2h-Ins, LDL-C, FFA, HOMA-IR, and AUCg, etc. but negatively correlated with HDL-C, adiponectin, and M-values. Serum GPHB5 levels did not change significantly during the OGTT, EHC, and lipid infusion. Physical activity and cold-exposure tests did not lead to changes in GPHB5 levels. GLP-1RA treatment resulted in a significant decrease in serum GPHB5 levels.

**Conclusions:**

GPHB5 may be a biomarker for MetS.

## Introduction

Metabolic syndrome (MetS) is a general term for a group of metabolic risk factors, including abdominal obesity, insulin resistance (IR), dyslipidemia, impaired glucose tolerance (IGT) and hypertension. It is an important phenotype leading to metabolic diseases such as coronary heart disease and type 2 diabetes mellitus (T2DM) (1, 2). The rapid increase in the prevalence of MetS and the resulting increase in the risk of cardiovascular and cerebrovascular diseases are worldwide public health problems (3, 4); affecting 25% of the adult population (5). In recent decades, researchers from various countries have done a lot of work on MetS, but its pathogenesis remains unknown, and there is a lack of effective biomarkers to predict its occurrence and development. Therefore, finding a reliable biomarker is very important for screening MetS and evaluating its prognosis.

The glycoprotein hormone family, including thyroid-stimulating hormone (TSH), follicle-stimulating hormone (FSH), and luteinizing hormone (LH), is involved in many aspects of physiological activity regulation, including reproduction, growth and development and energy metabolism (6, 7). Recent studies have found a new glycoprotein hormone, named glycoprotein subunit β5 (GPHB5) (8). GPHB5 has a similar structure to other glycoprotein hormones such as TSH, LH, and FSH, and its glycoprotein subunits have 30% homology (9, 10). Recombinant GPHB5 is a heterodimeric hormone that activates the TSH receptor (TSHR) *in vivo* and *in vitro* (11–13). GPHB5 was found to be expressed in the pituitary, retina, testis and skin and co-located with ACTH in the anterior pituitary, suggesting that it may be a new member of the anterior pituitary hormone family (12). However, the expression of GPHB5 is not completely consistent in the literature (9, 12, 14). The main sources of GPHB5 may be the brain, pituitary, testis and skin. In addition, an animal study showed that GPHB5 transgenic mice showed a phenotype of Grave’s disease in humans since GPHB5 served as an alternate ligand for the TSHR. Those mice had increased serum T4 levels and reduced body weight (12). Studies of gene intervention have shown that overexpression of GPHB5 leads to increased serum T4 levels, exophthalmos and weight loss in mice (12, 15), resulting in a similar effect on whole-body metabolism as TSH. However, there are few reports on the function and regulation of GPHB5 in humans. Especially in the occurrence and development of metabolic diseases, the role of GPHB5 is poorly understood.

Herein, we measure serum GPHB5 levels in MetS patients and normal controls and analyze their relationship with glucose and lipid metabolism and IR. We believe this is the first clinical study to explore the relationship between GPHB5 and MetS.

## Materials and Methods

### Participants

**Figure 1:**
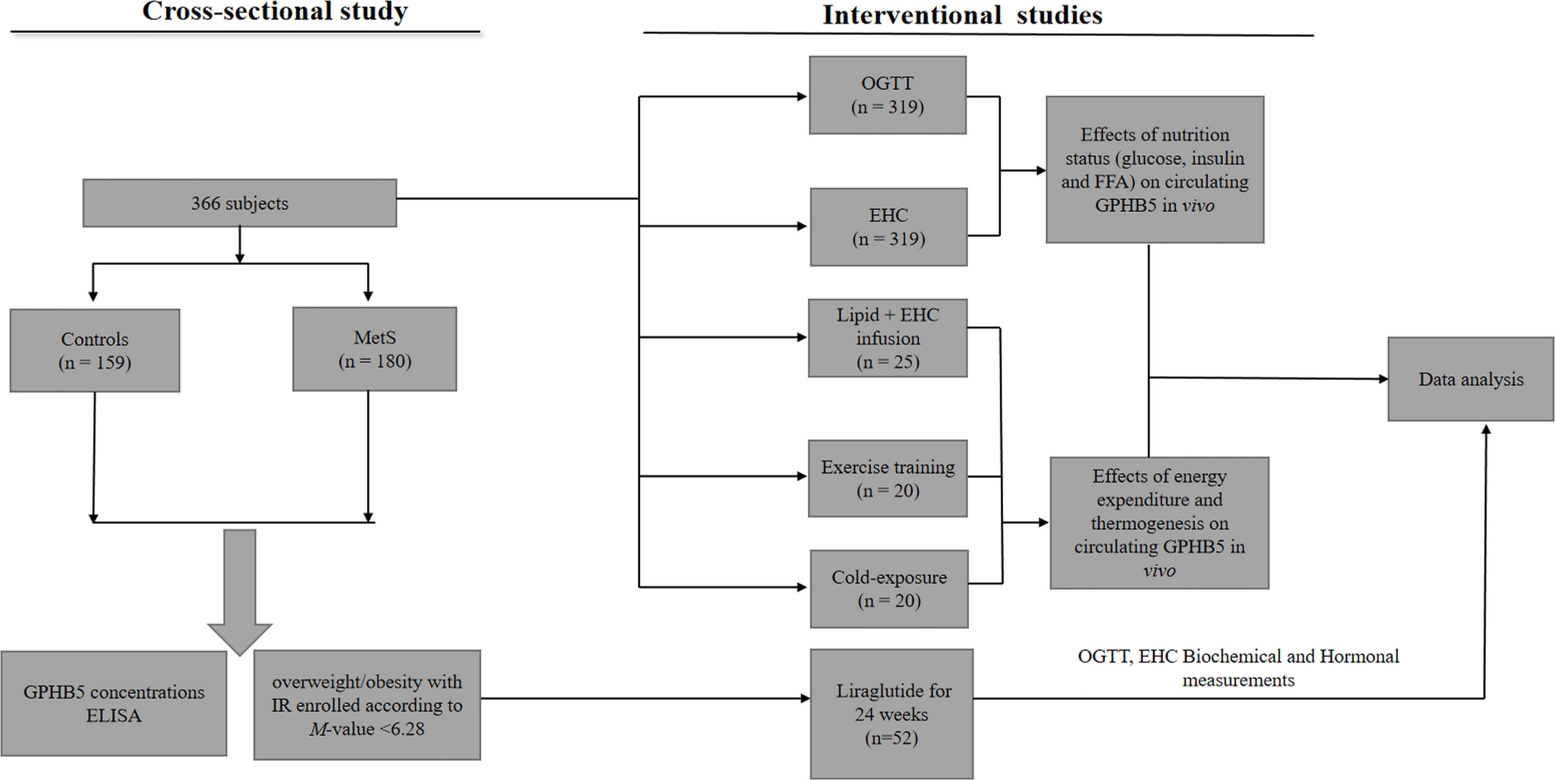
Clinical experimental design. OGTT, oral glucose tolerance test; EHC, euglycemic-hyperinsulinemic clamp; MetS, Metabolic syndrome.

A total of 366 subjects were included in this study, including 180 MetS patients with an average age of 26 (25–30) years and 159 healthy controls with an average age of 25 (24–28) years and 27 normal individuals [age 25 (24-29)] for intervention study. These participants were randomly selected from the 2017-2020 cross-sectional survey of obesity among young women in Chongqing, as a section of the Chongqing physique and health survey project. MetS was diagnosed based on Chinese Diabetes Society criteria (CDS guideline 2017) (16). MetS was diagnosed as follows: (1) abdominal obesity (waist circumference, WC ≥ 90 for male or ≥ 85 cm for female); (2) blood pressure (BP) ≥ 130/85 mmHg or receiving anti-hypertensive medication; (3) triglycerides (TG) ≥ 1.7 mmol/L. (4) high-density lipoprotein- cholesterol (HDL-C < 1.04 mmol/L). (5) fasting blood glucose (FBG) ≥ 6.1 mmol/L or 2-h blood glucose after the glucose challenge (2h-BG) ≥ 7.8mmol/L or known history of T2DM. Patients with the following diseases were excluded, such as heart failure, liver cirrhosis, liver and kidney failure, long-term use of steroids, cancer, infection, etc. Individuals were defined as normal glucose tolerance (NGT), impaired glucose tolerance (IGT), or T2DM according to WHO 1998 diagnostic criteria (17). In this study, MetS patients were diagnosed for the first time without any drug and lifestyle intervention. The control group was healthy subjects from physical examination, who had normal blood glucose, no family history of T2DM and hypertension, no clinical evidence of any disease, and did not take any drugs. The experimental design was shown in **Figure 1**. This study was approved by the Human Research Ethics Committee of Chongqing Medical University and has been registered on atchictr.org (ChiCTR1800019776).

### Anthropometric and Biochemical Measurements

Anthropometric measurements, including height, weight and blood pressure (BP), were performed at 8:00 am, and body mass index (BMI) was calculated. The waist circumference (WC) was determined as the minimum value between the iliac crest and the lateral costal margin. After 12 hr of overnight fasting, the blood sample was taken for biochemical measurements, including fasting blood glucose (FBG), blood glucose during oral glucose tolerance test (OGTT), glycosylated hemoglobin, insulin, triglyceride (TG), total cholesterol (TC), free fatty acid (FFA), HDL-C and LDL-C as described previously (17). The percentage of fat *in vivo* (FAT%) was examined using bioelectrical impedance (BIA-101; RJL Systems, Shenzhen, China). As previously reported, the homeostasis model assessment index (HOMA-IR) was calculated to assess IR (18).

### OGTT

OGTT was performed for study individuals at 8:00 am. after fasting for 12 h. Venous blood samples were drawn for GPHB5, adiponectin, glucose, and insulin (19).

### EHC Study

Euglycemic-hyperinsulinemic clamp (EHC) Test was performed on all subjects as previously described (20). Briefly, after overnight fasting, the test started at 8:00 am, and catheters were inserted into the left and right elbow anterior veins for collecting blood and infusing glucose and insulin. During the EHC, regular human insulin (1 mU/kg/min) was infused for two hours, and 20% glucose was infused at a variable rate to maintain blood glucose at fasting levels. The EHCs lasted for 120 min, and blood glucose was measured every 10 min. Glucose disposal rate (GDR) was defined as the glucose infusion rate (GIR) during the stable phase of EHC, which was related to the M-value. Venous blood was collected at 0, 80, 100, 110, and 120 min during the clamping and stored at - 80° C for insulin and GPHB5 determination.

### Lipid-Infused Experiment

A lipid-infusion experiment and the EHC were performed in 25 normal individuals (8 males and 17 females; age 25.84 ± 3.66 yr; BMI 21.17 ± 2.02 kg/m^2^) to establish a lipid-induced IR. Briefly, after 10-h of fasting, a polyethylene catheter was inserted into the anterior cubital vein at 8:00 am for the infusion of the glucose and insulin. Another catheter was inserted into the contralateral forearm vein for blood collection. Lipid and heparin (0.4 U/kg/min) were infused at a rate of 1.5 ml/min for 4 hours (21).

### Cold-Exposure Experiment

20 healthy individuals participated in the cold-exposure experiment, including 9 males and 11 females (age 25.95 ± 3.24 yr; BMI 21.77 ± 1.86 kg/m^2^). After 12-h fasting, individuals with light clothing were placed on a bed with a water-circulated cooling blanket (ThermoBlanket, P&C-AII, Hengbang Technology) at 8:00 am. The water temperature was regulated by electromyography. After 30 min of exposure to 27°C, a blood sample was drawn, and the water temperature was cooled to 18°C, and then reduced by 2°C every 3 min until 12°C. This temperature was maintained for 5 min, and then blood was drawn for GPHB5 measurement.

### Physical Exercise Test

20 young individuals 8 males and 12 females (age 26.16 ± 3.51 yr; BMI 21.50 ± 2.08 kg/m^2^) participated in the physical exercise test. After 12 hours of fasting, at 8:00 am, a treadmill exercise was performed on these individuals, with 60% of maximal oxygen consumption for 45 min. Blood samples were collected for the measurement of GPHB5 concentration at four-time points: baseline, after exercise, and rest for 60 and 120 min after exercise.

### GLP-RA Intervention Study

Fifty-two women with MetS (age 28.2 ± 4.3 yr) participated in the Glucagon-like peptide-1 receptor agonists (GLP-1RA, Lira) intervention study. All participants were given informed written consent about the side effects of Liraglutide (lira) at the onset of this study. All women did not use any drugs three months before enrollment. Women with a history of acute pancreatitis and thyroid tumors were excluded from this intervention study. Treatment was started after the basic evaluation, and the dose of the lira was increased from 0.6 mg to 1.8 mg/d sc once a day for 24 weeks. GTT, ITT, EHC, insulin, biochemical indexes, and GPHB5 levels were measured at the 12^th^ and 24^th^ weeks of treatment. All women underwent anthropometric measurements before and after GLP-1RA treatment. Blood samples were collected at 8:00 am on day 0, week 12, and week 24 of GLP-1RA treatment for GPHB5 and biochemical parameters measurement. Fasting blood samples were collected at 8:00 am on day 0, week 12, and week 24 of GLP-1RA treatment for GPHB5, insulin, and blood glucose measurement.

### GPHB5 and Adiponectin Measurement

Circulating GPHB5 was measured by an ELISA kit (Kete Biotechnology Co., Ltd, Jiangshu, China) following the manufacturer’s protocol. The sensitivity of this kit was 10 pg/ml, and intra- and inter-assay variations (CV) were both < 10%. The kit is highly sensitive and specific for the determination of human GPHB5 and is not interfered with by other cytokines. Serum adiponectin levels were also measured by an ELISA kit from Aviscerabio science Inc., USA (sk00010-01), with intra- and inter-assay CV of 4% - 8% and 8% - 12%, respectively, as previously reported (19).

### Bioinformatic Analysis

GPHB5 gene was mapped into the Search Tool or the Retrieval of Interacting Genes (STRING) database (v11.0) with a 0.4 confidence score to explore the protein-protein interaction (PPI) relationship between the hub gene and interacting genes. The adding nodes function of STRING was used to depict the third generation of relationship for the hub genes, which is the indirect relationship of the PPI network (22). Gene Ontology (GO) and Kyoto Encylopedia of Genes and Genomes (KEGG) analyses were performed using the clusterProfiler package. Gene classification was performed according to the biological processes (BP), cellular components (CC), and molecular functions (MF) from GO analysis. q-value < 0.05 indicates statistical significance.

### RT-PCR Analysis

GPHB5 mRNA expression was performed by RT-PCR assay, as previously described (20). Gene expressions were analyzed using the comparative threshold cycle (Ct) method in relation to the levels of the ß-actin. The sequences for GPHB5 were: F: 5’-CCAGACAG GTGACAGTGAAGC-3’ and R: 5’- ACATCGGACAGCCATAGGG-3’.

### Statistical Analysis

Data were expressed as mean ± SD or median with an interquartile range. All statistical analyses were performed by using the SPSS software package (version 2.0, SPSS Inc.). Two-tailed *p-value* < 0.05 was considered statistically significant. A Kolmogorov-Smirnov test was used to analyze whether each variable was normally distributed. Non-normal distribution data were logarithmically transformed before analysis. Linear regression analysis was used to evaluate the correlation between circulating GPHB5 and other indicators. Multiple regression analysis was performed to evaluate the statistically significant variables of circulating GPHB5. The quartile-quartile (Q-Q) plot was depicted using the R statistical package to show the expected distribution of *p* values in logistic regression analysis relative to the null hypothesis (http://www.r-project.Org). To provide a tool for identifying MetS patients using circulating GPHB5, we calculated the receiver operating characteristic (ROC) curve as an independent predictor and obtained a cut-off point value. Cochran-Armitage trend and Row Mean Scores tests were performed to analyze the tendency of serum GPHB5 concentrations associated with MetS. ROC curve analyses were performed to determine the value of using circulating GPHB5 to predict MetS. In the OGTT, the area under the glucose (AUCg) and insulin (AUCi) curve was determined according to the trapezoidal rule. The equation: $n={\left(\frac{Z_{\alpha /2}\sigma }{\varepsilon \mu }\right)}^{2}$ (σ, standard; µ, mean; Z_α/2_ = 1.96, α = 0.05, ε = 10%) was used for estimating sample size.^2^ In statistical analyses, *p* < 0.05 was considered significant.

## Results

### Bioinformatic Analysis for GPHB5-Related Genes and Signaling Pathways

**Figure 2:**
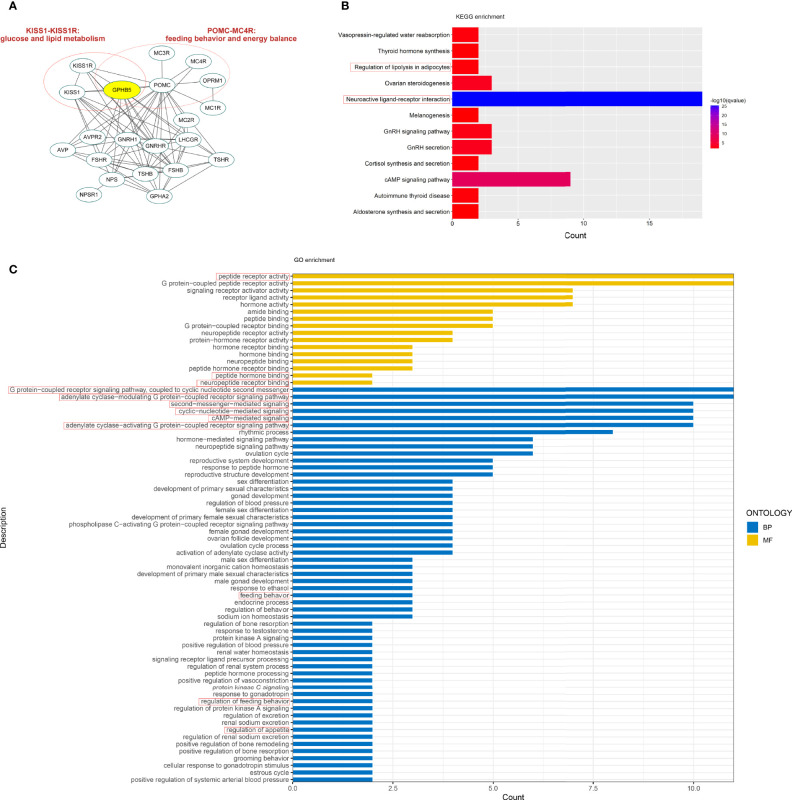
Bioinformatics analysis related to GPHB5. **(A)** PPI network. **(B)** KEGG enrichment. **(C)** GO analysis.

We initially conducted a bioinformatics investigation employing Internet data to understand the relationship between GPHB5 and metabolism. PPI network showed 11 genes interacting with GPHB5, including NPS, TSHB, TSHR, FSHR, POMC, LHCGR, GPHA2, AVP, GNRHR, and KISS1R, some of which are involved in metabolism and energy balance, such as POMC and KISS1R (23, 24). Further, we found ten more interacting proteins through additional functions (**Figure 2A**). KEGG analysis was performed with a q-value < 0.05. Twelve pathways were significantly enriched in KEGG analysis, including autoimmune thyroid disease, vasopressin- regulated water reabsorption, cortisol synthesis and secretion, GnRH secretion, aldosterone synthesis and secretion, regulation of lipolysis in adipocytes, thyroid hormone synthesis, melanogenesis, ovarian steroidogenesis, GnRH signaling, neuroactive ligand-receptor interaction, and cAMP signaling pathways, etc. Some are related to glucose and lipid metabolism and energy balance (**Figure 2B**). Using the human genome as a background variable (q-value < 0.05), the GO analysis annotated 56 BP and 16 MF pathways. Some pathways were related to feeding behavior and energy metabolism, such as OPRM1and melanocortin receptor family, etc. (**Figure 2C**). The results from bioinformatics analysis preliminarily suggest that GPHB5 may be associated with glucose and lipid metabolism and energy equilibrium.

### Distribution of GPHB5 mRNA Expression In Mouse Tissues

To identify the source of circulating GPHB5, we analyzed the expression of GPHB5 mRNA in tissues and organs in mice. We found higher expression in the heart, brain, liver, and skeletal muscle (**Figure S1**). Therefore, we believe that the main sources of GPHB5 may be the heart, brain, and liver.

### General Clinical Features and Serum GPHB5 Concentration in Study Individuals

**Figure 3:**
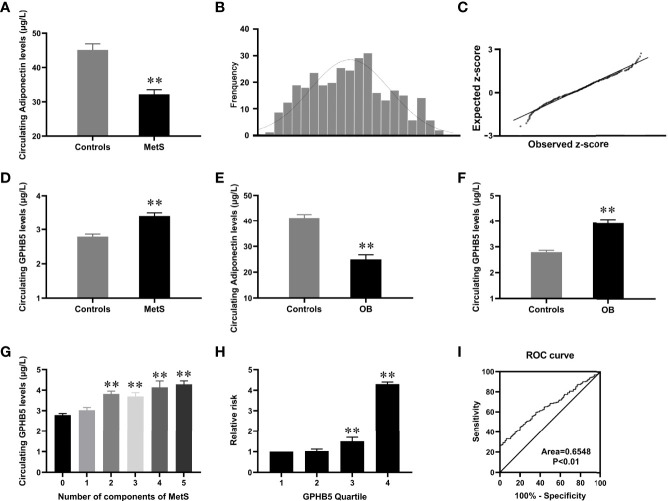
Circulating GPHB5 levels in the study population. **(A)** Circulating Adiponectin levels in healthy and MetS individuals. **(B)** Distribution of GPHB5 levels in healthy population. **(C)** Q-Q plot of normalized residuals versus a normal distribution. **(D)** Circulating GPHB5 levels in healthy and MetS individuals. **(E)** Circulating Adiponectin levels in lean and O/B individuals. **(F)** Circulating GPHB5 levels in lean and O/B individuals. **(G)** Circulating GPHB5 levels in relation to the number of MetS components. **(H)** The odds ratio of having MetS in different tertiles of circulating GPHB5. **(I)** ROC curve analysis of the prediction of MetS. O/B, overweight/obese. Data were means ± SD. **p < 0.01 *vs.* controls or tertile 1.

The general clinical characteristics of MetS women and control individuals are shown in **Table 1**. Compared with the control group, MetS women had higher BMI, WC, blood pressure (BP), TG, TC, LDL-C, FBG, two-hour blood glucose after glucose overload (2-h BG), fasting insulin (FIns), 2-h serum insulin after glucose overload (2 h-Ins), HOMA-_IR_, AUCi, AUCg, HbA1c, visceral adiposity index (VAI) and body adiposity index (BAI). However, HDL-C and M-value were lower in MetS patients. In addition, thyroid hormones (T3 and T4) and thyroid-stimulating hormone (TSH) were comparable in MetS patients and healthy controls (**Table S1**). As expected, serum adiponectin levels were significantly lower in MetS patients than in normal controls (**Table 1** and **Figure 3A**).

**Table 1:** Main clinical features and serum GPHB5 levels in all study population.

Characteristics	Overall (n = 339)	Controls (n = 159)	MetS (n = 180)
Age (years)	27 (25-31)	25 (24-28)	26 (25-30)
GPHB5 (µg/L)	3.16 (2.17-3.95)	1.89 (2.56-3.26)	3.44 (2.47-4.47)**
Adiponectin (µg/L)	37.4 (23.0-48.3)	42.8 (34.5-54.3)	32.6 (16.9-45.4)**
BMI (kg/m^2^)	22.1 (19.8-25.6)	20.2 (18.2-21.4)	24.4 (21.0-27.0)**
WC (cm)	76.0 (68.0-84.0)	69.0 (64.0-74.0)	80.0 (72.5-88.5)**
SBP (mmHg)	110 (102-119)	105 (99-111)	112 (104-122)**
DBP (mmHg)	72 (67-80)	71 (63-78)	74 (68-80)**
TC (mmol/L)	4.43 ± 1.00	3.95 ± 0.87	4.67 ± 0.98**
TG (mmol/L)	0.98 (0.69-1.61)	0.82 (0.65-1.06)	1.18 (0.70-1.96)**
HDL-C (mmol/L)	1.25(1.01-1.52)	1.42 (1.20-1.62)	1.17 (0.95-1.46)**
LDL-C (mmol/L)	2.58 (2.01-3.33)	2.11 (1.74-2.53)	2.89 (2.35-4.04)**
FFAs (mmol/L)	0.58 (0.41-0.88)	0.46 (0.33-0.67)	0.68 (0.46-1.02)**
FBG (mmol/L)	4.92 (4.47-5.35)	4.54 (4.31-4.89)	5.12 (4.58-5.54)**
2 h-BG (mmol/L)	6.00 (4.86-7.70)	5.26 (4.46-6.20)	6.94 (5.25-8.77)**
FIns (mU/L)	9.30 (6.60-18.0)	6.89 (5.57-8.70)	13.60 (7.47-22.30)**
2 h-Ins (mU/L)	61.9 (23.7-127.9)	34.5 (19.7-57.0)	97.0 (36.4-213.9)**
M-value (mg/kg/min)	7.03 (4.57-10.29)	9.49 (7.99-11.55)	5.26 (3.86-8.31)**
HOMA-_IR_	2.02 (1.33-4.11)	1.37 (1.12-1.83)	3.09 (1.52-5.37)**
AUCi (mU × h/L)	108.0 (69.0-180.0)	78.9 (49.4-104.2)	136.2 (84.2-222.6)**
AUCg (mmol × h/L)	10.3 (8.7-12.6)	9.1 (8.1-10.3)	11.5 (9.0-13.8)**
HbA1c (%)	5.2 (5-5.5)	5.1 (5-5.3)	5.2 (5-5.6)**
BAI	29.0 ± 3.90	26.5 ± 2.70	30.1 ± 3.91**
VAI	2.41 (1.53-4.06)	1.88 (1.39-2.57)	2.81 (1.76-4.89)**

Values are given as mean ± SD or median (Interquartile Range). MetS, Metabolic Syndrome; BMI, body mass index; WC, waist circumference; SBP, systolic blood pressure; DBP, diastolic blood pressure; FBG, fasting blood glucose; 2 h-BG, 2h blood glucose after glucose overload; FIns, fasting plasma insulin; 2 h-Ins, 2h serum insulin after glucose overload; M-value, the rate of glucose infusion; TG, triglyceride; TC, total cholesterol; HDL-C, high-density lipoprotein cholesterol; FFA, free fatty acid; HOMA-_IR_, homeostasis model assessment of insulin resistance; AUCg, the area under the curve of glucose during oral glucose tolerance test; AUCi, the area under the curve of glucose during insulin tolerance test; VAI, visceral adiposity index; BAI, body adiposity index. **p < 0.01 vs. Controls.

To investigate the distribution of circulating GPHB5 concentration, serum GPHB5 levels in 159 average women were measured by ELISA kit. The results showed that serum GPHB5 concentrations ranged from 1.35 to 5.12µg/L for 95% in the normal controls (**Figure 3B**). Q-Q plot showed that the error distribution was a standard normal distribution (**Figure 3C**). Interestingly, in contrast to low serum adiponectin levels, circulating GPHB5 concentrations were significantly elevated in MetS patients (**Table 1** and **Figure 3D**). Furthermore, obese/overweight (OB) individuals had lower adiponectin levels and higher GPHB5 levels than lean individuals (**Figures 3E, F**). These results indicated that GPHB5 might be related to metabolic disorders and obesity.

### Association of Serum GPHB5 Levels With Other Parameters and MetS in All Study Individuals

We performed linear and multivariate regression analyses to understand the relationship between GPHB5 and other indicators. Linear regression analysis revealed that circulating GPHB5 was significantly positively correlated with BMI, WHR, BP, FBG, 2 h-BG, FIns, 2 h-Ins, LDL-C, FFA, HbA1c, HOMA-IR, AUCi, AUCg, and BAI, while negatively correlated with HDL-C, M-value, and Adiponectin (**Table 2**). The dependent variable GPHB5 and other parameters (independent variables) were used for regression analysis to establish a multiple regression model. The results showed that HbA1c, AUCg, and BAI were independent influencing factors of serum GPHB5 (**Table S2**). The regression equation of gphb5 was: Y_GPHB5_ =-19.21 + 0.496× HbA1c + 0.96 × AUCg + 0.718 × BAI (R = 0.571, R2 = 0.326).

**Table 2:** Spearman correlation analysis of circulating GPHB5 and other indexes in all study population.

Variable	*r*	*p*
BMI (kg/m^2^)	0.471	< 0.01
WHR	0.28	< 0.01
SBP (mmHg)	0.362	< 0.01
DBP (mmHg)	0.197	< 0.01
FBG (mmol/L)	0.456	< 0.01
2 h-BG (mmol/L)	0.443	< 0.01
FIns (mU/L)	0.415	< 0.01
2 h-Ins (mU/L)	0.389	< 0.01
*M*-value (mg/kg/min)	- 0.533	< 0.01
TC (mmol/L)	0.322	0.84
TG (mmol/L)	- 0.131	0.078
HDL-C (mmol/L)	-0.15	< 0.05
LDL-C (mmol/L)	0.313	< 0.01
FFA (*μ*mol/L)	0.27	< 0.01
HbA1c	0.347	< 0.01
Adiponectin (µg/L)	- 0.485	< 0.01
HOMA-IR	0.414	<0.01
AUCi (mU × h/L)	0.355	< 0.01
AUCg (mmol × h/L)	0.46	< 0.01
VAI	0.049	0.216
BAI	0.39	< 0.01

p < 0.05, the data were statistically significant.

We further stratified the serum GPHB5 concentration according to the MetS components in MetS patients. Serum GPHB5 concentration is increased with the increased components of MetS (**Figure 3G**). Additionally, Cochran–Armitage trend and row mean score tests revealed that increased GPHB5 levels had a linear trend related to MetS (**Table S3**). To calculate the probability of MetS, serum GPHB5 levels were divided into four quartiles (1, < 2.23 µg/L; 2, 2.23-3.13 µg/L; 3, 3.13-3.92µg/L; 4, > 3.92 µ g/L). Logistic regression analysis revealed that odds ratios for developing MetS in the quartiles 3 and 4 were higher than the quartile 1 and 2 [95% confidence interval (CI) 3.08; 3.92µg/L for quartile 3 and 95% CI 3.69; 5.53µg/L for quartile 4 *vs.* quartile 1, both *p* < 0.01, **Figure 3H**].

To investigate whether GPHB5 can predict MetS, a ROC curve analysis was performed. The AUC of ROC was 0.65, the sensitivity was 79%, and the specificity was 60.6%, respectively (**Figure 3I**). The cut-off value for serum GPHB5 to predict MetS was 3.27µg/L.

### Effects of Blood Glucose and Insulin Levels on GPHB5 Concentration

**Figure 4:**
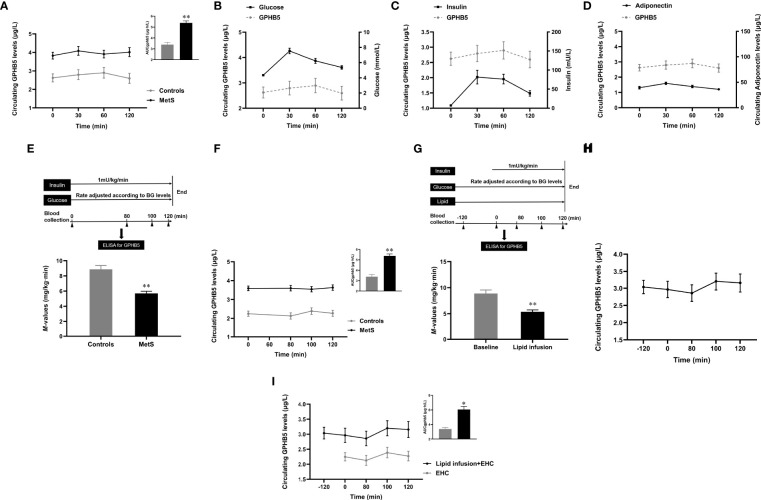
Circulating GPHB5 levels in OGTT, EHC and lipid-infusion studies. **(A)** Circulating GPHB5 levels and AUC_GPHB5_ in healthy and MetS individuals during the OGTT. **(B)** Circulating GPHB5 levels and blood glucose in healthy individuals during the OGTT. **(C)** Circulating GPHB5 levels and insulin in healthy individuals during the OGTT. **(D)** Circulating GPHB5 levels and adiponectin in healthy individuals during the OGTT. **(E)** EHC experimental design and M-value in healthy and MetS subjects. **(F)** During the EHC, GPHB5 levels and AUC_GPHB5_ in MetS and control individuals. **(G)** Experimental design of lipid-infusion + EHC (upper) and M-values (bottom) in healthy women. **(H)** Time course of GPHB5 levels changes during lipid-infusion + EHC. **(I)** Time course of GPHB5 levels changes during the EHC alone or lipid-infusion + EHC. AUC, area under the curve; BG, blood glucose; Data are means ± SD. *p < 0.05 or **p < 0.01 *vs.* controls.

To further understand the relationship between blood glucose, insulin, and GPHB5, we conducted OGTT and EHC experiments in MetS and healthy individuals. The results showed that serum GPHB5 levels did not change significantly during the OGTT in both normal controls and MetS individuals (from 3.43 ± 1.15 to 3.55 ± 1.35 µg/L at 30 min, and then to 3.51 ± 1.18 µg/L at 60 min, and finally to 3.48 ± 1.32 μg/L at 120 min), while AUC_GPHB5_ in MetS patients was significantly higher than that of controls (**Figure 4A**). During the OGTT, circulating GPHB5 levels did not change with blood glucose and insulin levels in normal individuals (**Figures 4B, C**). In addition, changes in circulating GPHB5 levels were similar to adiponectin in response to the glucose challenge (**Figure 4D**).

### EHC and Lipid-Infused Studies

To observe the effect of elevated insulin levels on GPHB5, we engaged in an EHC to increase insulin levels under normal blood glucose conditions (**Figure 4E** upper). Blood glucose was maintained at 4-6 mmol/L during the clamping, while insulin level increased from 12.4 ± 1.2 to 67.3 ± 5.4 mU/L. At the steady-states of the clamp, the M-values in MetS patients were significantly lower than those in the control group, suggesting that the whole-body insulin sensitivity in MetS individuals is reduced (5.69 ± 2.81 vs. 8.87 ± 2.46 mg/kg/min, *p <*0.01; **Figure 4E** bottom). Although serum insulin concentrations significantly increased during the clamping, circulating GPHB5 levels remained unchanged in MetS patients and controls compared with baseline (3.52 ± 1.29 *vs.* 3.43 ± 1.15 μg/L; **Figure 4F**). However, AUC_GPHB5_ was significantly higher in MetS individuals (5.61 ± 1.68 *vs.* 4.15 ± 1.36 μg·h/L; **Figure 4F**). These data indicate that GPHB5 may not be regulated by insulin *in vivo*.

To understand whether acute-increased FFA levels affect GPHB5 release, we performed a 4-h lipid-infusion combined with 2-h EHC in healthy individuals (**Figure 4G** upper). After 2-h lipid infusion, FFA concentration increased significantly from 0.46 ± 0.1 to 1.46 ± 0.2 mmol/L. As shown in **Figure 4G** (bottom), during the stable-state of the EHC, lipid-infusion significantly reduced M-values, suggesting that the acute increase of FFA resulted in an IR *in vivo*. However, 2-h lipid perfusion did not increase circulating GPHB5 concentration. At the beginning of EHC, elevated insulin levels led to a slight increase in GPHB5 concentration, but did not reach statistical significance (from 2.86 ± 1.21 μg/L at 80 min to 3.2 ± 1.26 μg/L at 100 min and then to 3.16 ± 1.34 μg/L at 120 min; **Figure 4H**). Compared with EHC alone, lipid-infusion did not change GPHB5 levels (**Figure 4I**). These results further suggest that GPHB5 may not be regulated by insulin and FFA.

### Effects of Cold-Exposure and Physical Activity on GPHB5 *In Vivo*

**Figure 5:**
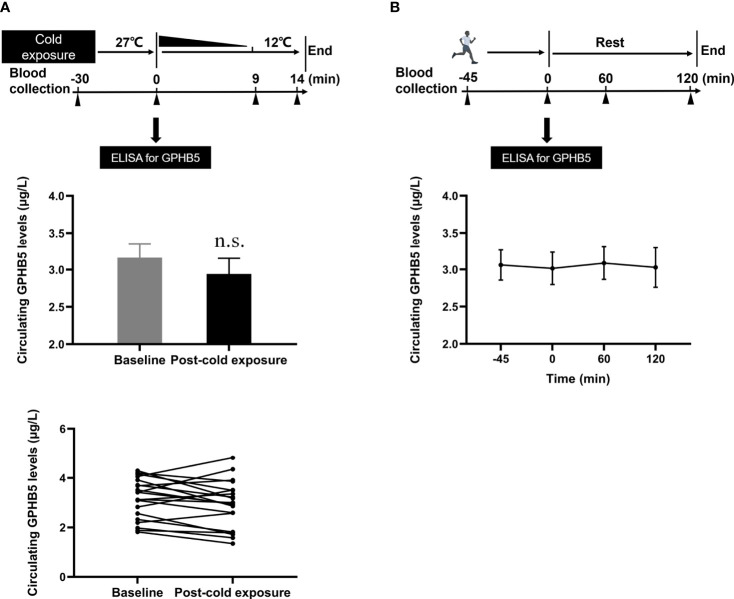
Circulating GPHB5 levels of physical activity and cold-exposure in healthy individuals. **(A)** Cold-exposure experimental design and circulating GPHB5 levels before and after cold-exposure. **(B)** Physical activity experimental design and circulating GPHB5 levels before and after exercise. Data are means ± SD. n.s., no significance; *p* > 0.05 vs. Baseline.

To understand the effects of thermogenesis and energy consumption on GPHB5 *in vivo*, we conducted cold-exposure (**Figure 5A** upper) and physical activity (**Figure 5B** upper) experiments in healthy subjects. During the cold-exposure investigation, we found that there were no significant changes in serum GPHB5 levels when the body temperature decreased from 27 °C to 12 °C (3.16 ± 0.81 *vs.* 2.94 ± 0.95 μg/L; **Figure 5A** bottom). Furthermore, there was no significant change in serum GPHB5 level before and after physical activity (3.01 ± 0.98 *vs.* 3.06 ± 0.91μg/L; **Figure 5B** bottom). These data show that short-term thermogenic effects and energy consumption do not regulate the secretion and release of GPHB5 *in vivo*.

### GLP-1RA Therapy for 24 Weeks Reduced Circulating GPHB5 Levels in MetS Patients

**Figure 6:**
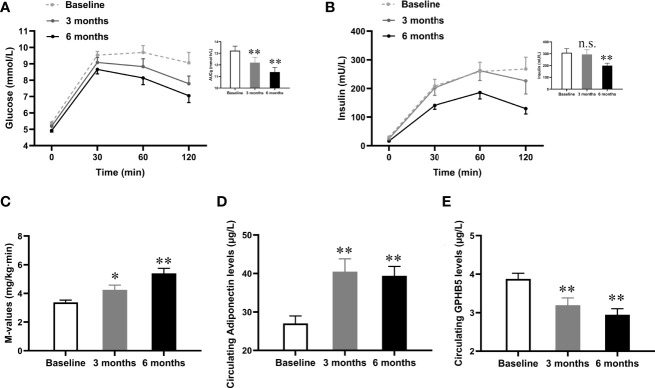
Effects of GLP-1RA treatment on serum GPHB5 levels and insulin sensitivity in MetS women. **(A)** Blood glucose levels and AUCg during the OGTT at pre- and post-treatment. **(B)** Insulin levels and AUCi during the OGTT at pre- and post-treatment. **(C)** M-values during the EHC at pre- and post-treatment. **(D)** Serum adiponectin levels at pre- and post-treatment. **(E)** Serum GPHB5 levels at pre- and post-treatment. AUC, area under the curve during oral glucose tolerance. Data are means ± SD. *p < 0.05 or **p < 0.01 as compared with Baseline. n.s., no significance; p > 0.05 vs. Baseline.

To investigate the impact of improved insulin sensitivity on GPHB5 levels, we used Lira therapy for 24 weeks in 70 MetS patients. After GLP-1RA intervention for 6 months, BMI, WHR, Fat %, BP, TG, TC, LDL-C, FFA, FBG, 2-h BG, FIns, 2-h Ins, HOMA-_IR_, BAI, and VAI in MetS patients decreased significantly compared with those before treatment, while HDL-C was increased in these patients (**Table 3**). In addition, glucose tolerance was significantly improved after treatment (**Figures 6A, B**). As expected, the M-values of the clamp after treatment in MetS women increased significantly compared with that before treatment (**Table 3** and **Figure 6C**). These data indicate an improved insulin sensitivity *in vivo.* Importantly, after GLP-1RA treatment, serum adiponectin levels in MetS women increased significantly (**Table 3** and **Figure 6D**), while serum GPHB5 levels decreased significantly (**Table 3** and **Figure 6E**).

**Table 3:** Main clinical and metabolic features pre- and post-treatment with GLP-1RA in obese individuals with IR.

Variable	Baseline	3 months	6 months
GPHB5 (µg/L)	3.88 ± 0.85	3.17 ± 0.96**	2.94 ± 0.86**
Adiponectin (µg/L)	25.9 (21.1- 30.1)	36.6 (30.6 - 44.4)**	39.4 ± 13.3**
BMI (kg/m^2^)	28.7 ± 3.70	26.3 ± 3.64**	25.4 ± 3.32**
WC (cm)	93.6 ± 9.15	86.9 ± 9.60**	84.5 ± 8.77**
WHR	0.90 ± 0.46	0.88 ± 0.57**	0.86 ± 0.51**
FAT (%)	41.9 ± 7.38	35.86 ± 5.33	36.1 ± 5.17**
SBP (mmHg)	117.4 ± 10.9	115.6 ± 12.7	112.9 ± 12.9
DBP (mmHg)	73.1 ± 9.2	75.1 ± 8.5	73.3 ± 9.0
TC (mmol/L)	4.79 ± 0.75	4.40 ± 0.82	4.23 ± 0.90**
TG (mmol/L)	1.68 (1.50-2.32)	1.52 (0.98-2.22)	1.08 (0.80-1.84)**^▲^
HDL-C (mmol/L)	1.17 ± 0.25	1.11 ± 0.25*	1.28 ± 0.31
LDL-C (mmol/L)	2.80 ± 0.71	2.56 ± 0.52*	2.37 ± 0.67**
FFA (µmol/L)	0.45 ± 0.17	0.41 ± 0.18*	0.40 ± 0.14
FBG (mmol/L)	5.40 ± 0.43	5.13 ± 0.33	4.92 ± 0.46**
2 h-BG (mmol/L)	8.29 (7.30-9.64)	7.53 (5.91-9.53)	6.36 (5.80-7.97)**
FIns (mU/L)	30.3 ± 15.8	22.9 ± 10.9*	16.3 ± 11.0**
2 h-Ins (mU/L)	178.3 (130.2 -273.8)	166.1 (97.7-253.2)	108.2 (72.4-132.0)**
M-value (mg/min/kg)	3.32 (2.88-4.03)	3.88 (3.45 - 4.42)*	4.65 (3.99-6.53)**
HOMA-_IR_	7.39 ± 4.12	5.26 ± 2.65	3.61 ± 2.56**
AUCi	249.6 (178.1-399.2)	254.8 (168.2-331.5)*	165.9 (124.3-265.1)**
AUCg	13.2 ± 2.3	12.2 ± 2.2	11.4 ± 2.2**
BAI	33.3 ± 3.3	30.1 ± 3.5**	31.0 ± 3.3**
VAI	3.11 (1.94 - 4.00)	2.98 (1.73-4.09)	1.56 (1.16-2.87)**^▲^

Values were given as mean ± SD or median range. *p < 0.05; **p < 0.01 vs. Baseline; ^▲^p < 0.05.

## Discussion

The increase in obesity, especially adolescent obesity, is considered to be a serious public health problem. More and more evidence shows that there is an important relationship between obesity and MetS (25, 26). Genetic background and environmental factors such as diet play a crucial role in the effluence of MetS. Recently, some cytokines/peptide hormones were found to play an important role in the pathogenesis of MetS (27, 28). To exclude the effects of gender and age-related IR. young women with and without MetS were selected to participate in the cohort study.

In this study, we first discovered the connection between GPHB5 and metabolic disorders through bioinformatics analysis. We found that in contrast to circulating adiponectin levels, circulating GPHB5 levels were markedly increased in MetS women. Circulating GPHB5 levels were significantly correlated with obesity, glucose and lipid metabolism, and IR. In overweight/obese individuals, circulating GPHB5 levels were significantly increased. Because GPHB5 and TSH have similar structures and functions, and can combine with TSHR (29), we speculate that GPHB5 may have some effects similar to TSH, such as the effect on glucose and lipid metabolism, fat storage and distribution. Therefore, this helps to explain the elevated GPHB5 levels in overweight/obese individuals. These preliminary results reveal that GPHB5 may be related to the pathogenesis of MetS. In addition, we found that participants with two MetS components had similar GPHB5 levels with MetS participants, indicating that any characteristic related to MetS could lead to an increase in circulating GPHB5 levels. Therefore, GPHB5 may be a biomarker related to all metabolic disorders. To our knowledge, this is the first clinical cohort study to explore the association of GPHB5 and metabolic disease.

To explore the effects of nutritional status and hormone levels on serum GPHB5, we conducted a series of interventional studies. We found that elevated blood glucose and insulin levels caused by an oral glucose challenge did not cause significant changes in circulating GPHB5 in normal control or MetS women. This result suggests that circulating GPHB5 may not be regulated by blood glucose and insulin, or that blood glucose and insulin reverse regulate GPHB5 and thus counteract their respective effects.

To examine the impacts of glucose and insulin on GPHB5 we conducted an EHC study on MetS and healthy women. We found that elevated insulin levels did not affect circulating GPHB5 levels when blood glucose was maintained at basal levels. In the clamp study of large samples, we found that there was a significant negative correlation between the GPHB5 level and M-value. Therefore, it is suggested that high GPHB5 levels are related to reduced insulin sensitivity. These results are not consistent with a previous animal study, which found that elevated GPHB5 decreased blood glucose and blood lipid and insulin levels, suggesting that GPHB5 is related to blood glucose, insulin and blood lipid levels. The cause of this conflict is not clear but may be related to experimental conditions or a difference in species. However, based on the results of a variety of intervention studies in humans, we believe that the secretion and release of GPHB5 may not be regulated by short-term elevations in blood glucose and insulin.

Many studies have shown that elevated plasma FFA can inhibit glucose transport and phosphorylation causing acute-IR *in vivo* (30, 31). Therefore, we conducted a lipid infusion combined with an EHC experiment. Lipid-infusion-induced serum FFA increase and EHC hyperinsulinemia did not cause a significant increase in circulating GPHB5 concentration. These findings suggest that GPHB5 secretion may be unaffected by short-term serum FFA and insulin levels. However, whether GPHB5 in T2DM is affected by IR-induced long-term hyperinsulinemia and high FFA levels remains unclear, and further research is needed.

It has been reported that acute exercise can increase some circulating factors and hormones such as FFAs, glucocorticoid cortisol, angiopoietin-like protein 4 (ANGPTL4) and follistatin-like 1 (FSTL-1), etc (21, 32). Therefore, we assessed the effect of acute exercise on serum GPHB5 levels in healthy individuals. A 45-min treadmill exercise did not alter serum GPHB5 levels. We, therefore, believe that the metabolic state of skeletal muscle does not affect the secretion or release of GPHB5.

Cold-exposure has been considered as a potential treatment for metabolic diseases by promoting brown adipose tissue (BAT) activity and thermogenesis (33). In the present study, we look at whether circulating GPHB5 levels were affected by cold-exposure. Our results show that cold-exposure did not affect circulating levels of GPHB5. We speculate that the secretion of GPHB5 may not be regulated by changes in BAT metabolism.

Liraglutide is a typical representative of GLP-1RA. GLP-1-RA has been reported to inhibit food intake, reduce body weight, and reduce blood glucose by promoting insulin secretion and improving glucose and lipid metabolism as well as IR (34, 35). These drugs have been widely used in the therapy of T2DM and as a weight-loss drug (36). In fact, GLP-1RA treatment has been reported to have significant benefits for all met components (37). In our MetS subjects, six months of GLP-1 therapy led to body weight loss, improved glycol-lipid metabolism and IR. In addition, the insulin sensitizer, adiponectin, showed levels that were significantly elevated after GLP-1-RA treatment. With these changes and improvements in insulin sensitivity, circulating GPHB5 levels gradually decreased after 3 to 6 months of treatment. These results further suggest that GPHB5 is associated with impaired glucose-lipid metabolism and IR, and is a negative regulator. The more severe the IR, the more GPHB5 is secreted and released, and the higher its serum concentration. Overall, our data fully demonstrate that GPHB5 is involved in glucose and lipid metabolism and IR, and is mainly regulated by the metabolic state of the liver. Therefore, it may be a hepaticytokine. However, the underlying mechanism of the relationship between elevated GPHB5 and MetS and the exact contribution to the pathogenesis of MetS remains to be further studied.

Our study also has some limitations: 1) as a cross-sectional study, the current results can not clarify the causal relationship between GPHB5 and MetS; 2) The population included in this study was Han nationality. Thus, caution should be taken in extending the data of our study to other races; 3) the GLP-1RA intervention study is open and non-randomized, which has a certain impact on the analysis of results.

In summary, the present study explores the relationship between GPHB5 and metabolic diseases for the first time. The results show that the level of circulating GPHB5 levels was significantly increased in MetS subjects, which was significantly related to MetS components, such as obesity, hyperglycemia, hyperlipidemia and blood pressure. Therefore, GPHB5 may be a biomarker for predicting and diagnosing MetS, In addition, GPHB5 may become a drug target to benefit MetS patients.

## Data Availability Statement

The original contributions presented in the study are included in the article/**Supplementary Material**. Further inquiries can be directed to the corresponding authors.

## Ethics Statement

This study was approved by the Human Research Ethics Committee of Chongqing Medical University and has been registered on atchictr.org (ChiCTR-occ-11001422). The patients/participants provided their written informed consent to participate in this study.

## Author Contributions

TX, QL and LL collected and analyzed these data. SZ engaged in bioinformatics analysis. HL and CC critically reviewed and edited the manuscript. GY and MY, the guarantor of this work and, as such, had full access to all of the data in the study and takes responsibility for the integrity of the data and the accuracy of the data analysis. All authors contributed to the article and approved the submitted version.

## Funding

This work was supported by research grants from Natural Science Foundation Project of Chongqing CSTC (cstc2015jcyjA10084), Science and Technology Program of Health Bureau of Chongqing (2016MSXM083 and 2017MSXM20) and Chongqing graduate scientific research innovation project (CYS21221). The founders have no roles in study design, data collection, data analysis, interpretation or writing of this research.

## Conflict of Interest

The authors declare that the research was conducted in the absence of any commercial or financial relationships that could be construed as a potential conflict of interest.

## Publisher’s Note

All claims expressed in this article are solely those of the authors and do not necessarily represent those of their affiliated organizations, or those of the publisher, the editors and the reviewers. Any product that may be evaluated in this article, or claim that may be made by its manufacturer, is not guaranteed or endorsed by the publisher.

## Abbreviations

GPHB5, glycoprotein subunit β5; EHC, euglycemic-hyperinsulinemic clamps; OGTT, oral glucose tolerance tests; GO, gene ontology; MCR, melanocortin receptor; OPRM1, μ-opioid receptor; POMC, Pro-opiomelanocortin; KEGG, Kyoto Encylopedia of Genes and Genomes; FBG, fasting blood glucose; 2 h-BG, 2-hour blood glucose after glucose overload; FIns, fasting insulin; 2h-Ins, 2-hour plasma insulin after glucose overload; LDL-C, low-density lipoprotein cholesterol; FFA, free fatty acid; HOMA-IR, homeostasis model assessment of insulin resistance; AUCg, area under the glucose.
