# Supplementary material for: GPHB5 Is a Biomarker in Women With Metabolic Syndrome: Results From Cross-Sectional and Intervention Studies
Source: Front Endocrinol (Lausanne). 2022 Jun 9;13:893142. doi: 10.3389/fendo.2022.893142 (PMC9218212; doi:10.3389/fendo.2022.893142)
Supplement: Supplementary file 1 [file DataSheet_1.docx]

**SUPPLEMENTARY MATERIAL**

**TABLE S1** Thyroid hormone levels in MetS patients and healthy controls

| variable | Controls | MetS | P-value |
| --- | --- | --- | --- |
| T3 (pmol/L) | 4.60 (3.70 - 5.00) | 4.80 (4.22 - 5.18) | 0.483 |
| T4 (pmol/L) | 15.20 (13.40 - 16.30) | 13.75 (12.58 - 17.62) | 0.425 |
| TSH (μIU/ml) | 2.12 (1.54 - 2.79) | 2.58 (1.92 - 5.80) | 0.109 |

TSH, Thyroid stimulating hormone. Data are median with interquartile range. **p* < 0.05 or ***p* < 0.01 as compared with Baseline.

**TABLE S2** Multiple regression analysis of the association of GPHB5 and other indexes in all study subjects.

| **Variable** | **B** | **SD** | **β** | **t** | **P** | **VIF** | **R** | **R2** | **Adjusted R2** |
| --- | --- | --- | --- | --- | --- | --- | --- | --- | --- |
| HbA1c | 0.496 | 0.225 | 0.15 | 2.21 | < 0.05 | 1.242 | 0.571 | 0.326 | 0.303 |
| AUCg | 0.960 | 0.290 | 0.25 | 3.31 | < 0.001 | 1.523 |  |  |  |
| BAI | 0.718 | 0.212 | 0.24 | 3.38 | < 0.001 | 1.264 |  |  |  |

*VIF, variance inflation factor; ß, standardized regression coefficients; SD, standard deviation; AUCg, the area under the curve of glucose during oral glucose tolerance test; BAI, body adiposity index.*

**TABLE S3** Row Mean Scores and Cochran-Armitage Trend test of the impact of circulating GPHB5 levels on MetS.

|  | **MetS** | |
| --- | --- | --- |
| **Model adjusted** | **χ^2^** | ***p-*value** |
| Row Mean Scores test | 316.876 | < 0.0001 |
| Cochran-Armitage Tread Test | 21.313 | < 0.0001 |

**
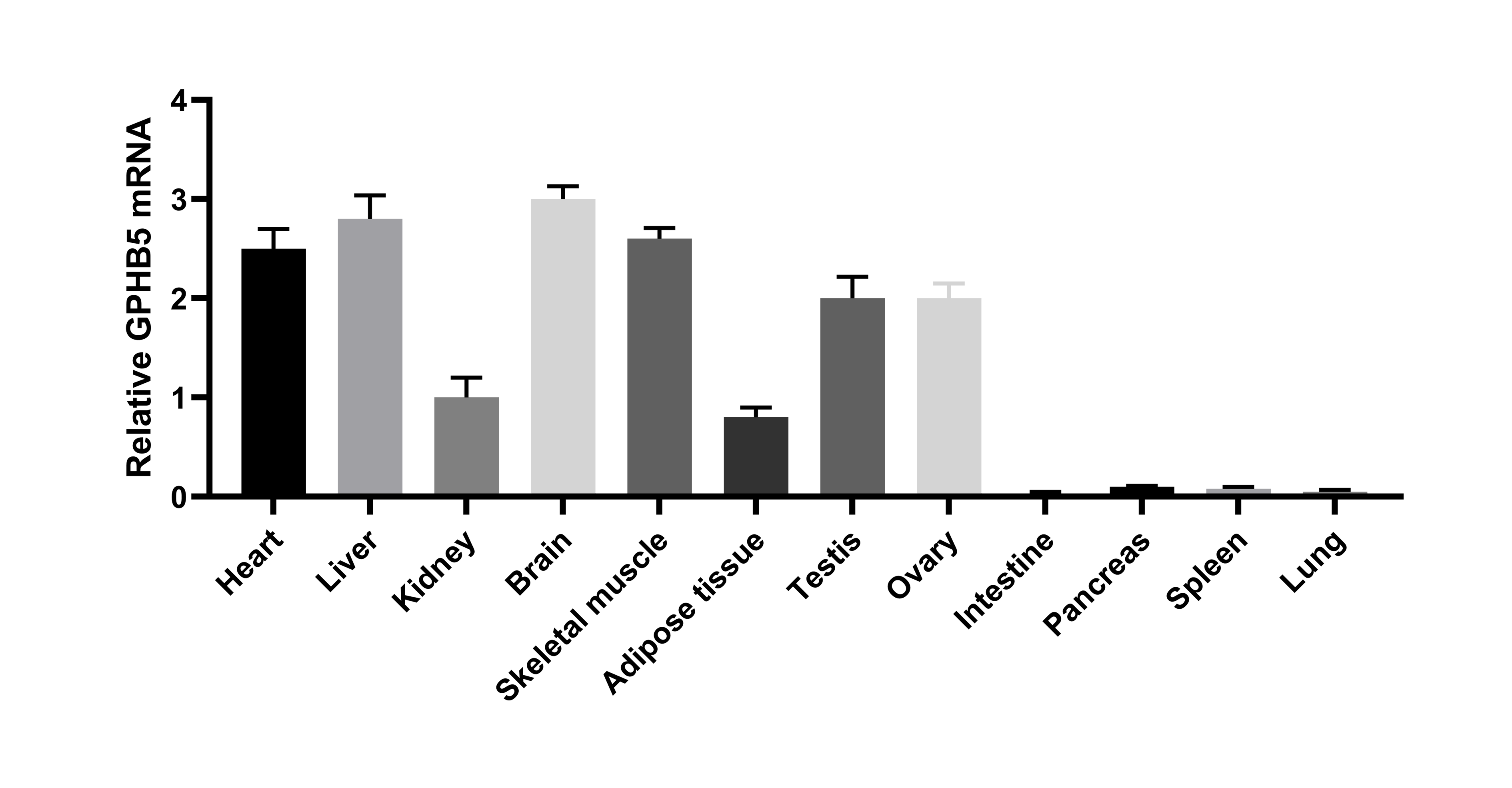
**

**Figure S1** Distribution of GPHB5 mRNA expression in C57BL/6J mouse tissues. GPHB5 expression determined by RT-PCR. (n = 3 per group for in vivo analysis)
